# No Association between Clinical Periodontal Conditions and Microbiological Findings on Driveline of Patients with Left-Ventricular Assist Devices (LVAD)

**DOI:** 10.3390/antibiotics10101219

**Published:** 2021-10-07

**Authors:** Gerhard Schmalz, Sven-Paul Zöbisch, Jens Garbade, Josephine Rast, Mirjam Eisner, Justus Wagner, Tanja Kottmann, Christian Binner, Sandra Eifert, Dirk Ziebolz

**Affiliations:** 1 Department of Cariology, Endodontology and Periodontology, University of Leipzig, 04103 Leipzig, Germany; gerhard.schmalz@medizin.uni-leipzig.de (G.S.); spzoebisch@googlemail.com (S.-P.Z.); rast.josephine@gmx.de (J.R.); mirjamcharlotte@web.de (M.E.); justus7@gmx.de (J.W.); 2 Department of Cardiac Surgery, Klinikum Links der Weser, 28277 Bremen, Germany; Jens.Garbade@medizin.uni-leipzig.de; 3 CRO Dr. med. Kottmann GmbH & Co. KG, 59077 Hamm, Germany; tk@cro-kottmann.de; 4 Department of Cardiac Surgery, Heart Center Leipzig, 04289 Leipzig, Germany; Christian.Binner@leipzig-heart.de (C.B.); sandra.eifert@helios-gesundheit.de (S.E.)

**Keywords:** dental care, left ventricular assist device, periodontitis, microbiology

## Abstract

The aim of this retrospective study was to investigate whether there would be an association between periodontal disease parameters and positive bacterial findings at the driveline of patients with a left ventricular assist device (LVAD). Patients with an LVAD, who underwent a full oral and microbiological examination between 2016 and 2018, were included. During oral examination, periodontitis severity (stage and grade) and the periodontal inflamed surface area (PISA) were evaluated. A microbiological analysis was performed from swabs of the driveline, whereby different bacterial species were cultivated and analyzed. A total of 73 patients were included in the current study. The majority of participants (80.8%) had at least one positive bacterial finding during the study period. Most patients had a periodontitis stage of III-IV (80.9%). The determined PISA of the total group was 284.78 ± 352.29 mm^2^. No associations were found between the periodontal disease parameters and the bacterial findings in general, the bacterial findings on the day of oral examination or the bacterial findings 12 months prior to/after the oral examination (*p* > 0.05). Periodontitis is not associated with cultivated microbiological findings at the driveline of patients with an LVAD and thus appears not to be a risk indicator for driveline colonization. Nevertheless, the high periodontal burden in LVAD patients underlines the need for their improved periodontal care.

## 1. Introduction

To unload the failing left ventricle during the treatment of severe heart failure (HF), left-ventricular assist devices (LVAD) are commonly used to maintain end-organ perfusion and improve functional capacity [1]. In Germany, the rate of VAD implantation has been approximately 900 per year in recent years. Due to technical, surgical and medical improvements during the past decade, both the number of patients living on LVAD and their life expectancy have increased [1]. While LVAD as a life-saving therapy measure leads to an improved patient outcome, several device-specific complications can occur, including infections and coagulation-related problems such as thrombosis, bleeding or device malfunction [2]. Until now, infection following VAD implantation has been particularly relevant to LVAD carriers because there is no full implantable device option. The system contains a driveline connecting the implanted pump device to the energy and leads through the skin in a subfascial tunnel. As this is an unnatural exit site, it represents a potential risk of microbial invasion [3]. Specific surgical techniques were developed to lower the rate of infection.

Driveline infections are common and sometimes difficult to manage, often requiring a prolonged antibiotic treatment [3,4,5,6]. Recent observational studies showed a prevalence of up to 30% of driveline infections during the first months after LVAD implantation [3,5]. Thus, the most often related bacteria are reported to be *Staphylococcus aureus*, coagulase-negative *Staphylococci*, *Enterobacteriaceae* and *Pseudomonas aeruginosa* [3,5]. Moreover, several risk factors for a driveline infection of LVAD systems are discussed, including system-specific issues (biofilm) as well as indication for LVAD [5], device design and LVAD-induced immune system dysfunction and immunocompromised status of HF patients in general [7], or co-morbidities [3,8]. Therefore, co-morbidities such as diabetes mellitus, which may be amplified under continuous blood flow, and high body mass index can be discussed [8].

Patients suffering from severe HF who are treated by LVAD have been found to show insufficient oral health conditions, especially a high prevalence of periodontal diseases and an enormous need for periodontal treatment [9,10]. Overall, the dental management of these severely diseased patients is difficult [11,12]. It is known that individuals with advanced periodontal diseases and/or inflammation have a high risk of bacteremia during dental interventions and ordinary procedures such as tooth brushing or interdental flossing, making the oral cavity and especially the inflamed periodontal pocket an important entry point for potentially pathogenic bacteria [13,14]. Thus, different potential mechanisms (direct and indirect) were described to explain the causal relationship of periodontal inflammation and systemic effects, e.g., on chronic heart disease. In this context, bacteremia related to oral inflammation appears to be a key problem [15]. Against this background, the potential role of periodontal inflammation as a risk factor for the development of LVAD-related infections appears to be a plausible option. Only one previous study examined a potential relationship between (history of) driveline infection and oral health conditions and failed to confirm a relationship [9]. However, no previous study has investigated whether there would be an association between periodontal disease severity as well as inflammation and positive microbiological findings at the driveline of LVAD. It might be conceivable that periodontal inflammation as a chronic infectious condition with a complex microbiological and immunological pathogenesis [15,16] might indicate an increased risk for LVAD-related infectious complications. This might also be of relevance for prevention and the antibiotic treatment of patients.

Accordingly, the aim of this study was to detect whether there would be an association between positive bacterial findings at the driveline and periodontal parameters. It was hypothesized that periodontal disease severity and inflammation would be associated with bacterial findings and, therefore, a potential risk indicator for driveline colonization.

## 2. Results

### 2.1. Patients

A total of 73 patients out of 128 individuals screened for eligibility, with a mean age of 60.92 ± 8.98 years, were included in the current study. About one-third (30.1%) had a driveline infection during the examination period. The average time since LVAD implantation at the time point of oral examination was 32.00 ± 24.03 months. An overview of the general and LVAD-related parameters is shown in Table 1.

### 2.2. Microbiological Findings

The majority of participants, i.e., 80.8% had at least one positive (cultured) bacterial finding during the study period at the entry point of the driveline. Thus, 46.6% of all patients had a bacterial finding on the day of the oral examination, while 63% of participants had a positive microbiological finding within 12 months prior to/after oral examination. The most often detected species were *Staphylococcus aureus* (16.4%) and *Staphylococcus epidermidis* (16.4%; Table 2).

### 2.3. Oral Examination

The included patients had on average 18.99 ± 7.58 remaining teeth. The majority of participants had a periodontitis stage III-IV (80.9%). The determined PISA of the total group was 284.78 ± 352.29 mm^2^ (Table 3).

### 2.4. Associations between Microbiology and Oral Findings

Comparing patients with and without any bacterial finding in the study period, no differences in periodontal parameters were found, indicating no associations between the periodontal parameters and bacterial findings (*p* > 0.05; Table 4). Similarly, no statistically significant differences regarding periodontal parameters were detected between patients with and without bacterial findings on the day of examination (*p* > 0.05; Table 5) and in the 12 months prior to/after oral examination (*p* > 0.05; Table 6), respectively.

## 3. Discussion

The included patients with an LVAD showed a high prevalence of periodontitis. No associations between periodontal parameters (severity and inflammation) and (cultured) microbiological findings at the LVAD driveline were confirmed.

This is the first study that examined potential associations between periodontal disease and bacterial findings at the driveline of patients with an LVAD. Accordingly, a comparison with the available literature is difficult. While the previous study failed to confirm a relationship between driveline infection and oral health [9], the current study also failed to confirm an association between positive bacterial findings at the driveline and periodontal parameters; however, the patient cohort in the current study was based on the same collective of patients as the previous study [9]. Therefore, the hypothesis that periodontitis is a risk indicator for microbiological colonization of the driveline and potentially driveline infections must be refused at this point. Periodontitis is a polymicrobial, multifactorial disease, which is characterized by microbiological dysbiosis and immunological disbalance [17]. The supposed potentially periodontal pathogenic bacteria are Gram-negative, anaerobic bacteria, e.g., *Prophyromonas gingivalis*, *Tannerella forsythia* or *Treponema denticola* [18]. However, the oral cavity hosts a high variety of bacteria, including other species such as *Staphylococcus aureus* or *Pseudomonas aeruginosa*, whereby the periodontal biofilm can be a source for dissemination of these bacteria [19,20]. Interestingly, some species, including *Staphylococcus aureus*, appear predominant in healthy periodontal conditions [19]; this might explain the absence of an association between such infections and periodontal inflammation. Generally, the finding that the most frequently detected bacteria in this current study were *Staphylococci* is in line with previous studies [3,5]. Both of the most commonly detected species, i.e., *Staphylococcus aureus* and *Staphylococcus epidermidis*, are commensal microbiota on epithelial surfaces [21,22]. The skin around the driveline thereby appears to be a more probable source for colonization than the oral cavity. However, it was not the aim of this current study to investigate whether oral bacteria are the cause of microbiological findings at the driveline, but instead whether periodontal inflammation could be a risk indicator for driveline colonization.

To prove whether- potential periodontal pathogens could be causal for LVAD-related infections, other localizations and the examined bacteria would need to be investigated. First, the above-mentioned bacteria related to periodontitis are anaerobic, showing a complex metabolism and special cultivation requirements [23]. Second, the common potential periodontal pathogens or their DNA were found in different cardiac tissue [24,25]. Furthermore, the causality of such an infection would be hard to prove; therefore, a study on the oral cause of LVAD-related infection would need another design similar to the current study. Regardless, the clinical consequence of such a study remains questionable for the following reasons: on the one hand, periodontal diseases need to be treated in any patient suffering from them, irrespective of cardiac disease. On the other hand, it is well documented that poor periodontal health increases the risk of poor cardiovascular outcomes. Thus, improving oral hygiene and reducing oral inflammation should be fostered to reduce the incidence of bacteremia of oral origin in any way. In this context, the high periodontitis prevalence in the current study needs to be discussed. In comparison to the Fifth German Oral Health Study (DMS V), a representative study for the German general population [26], the prevalence of periodontitis was remarkably high in the current study. This is comparable to other studies with severely heart diseased individuals [9,27,28,29,30]. This highlights a healthcare gap that has already been addressed previously [10]. However, this high prevalence might blur the potential of periodontal disease as a risk indicator because only a minority of patients were periodontally healthy or mildly diseased, respectively. To circumvent this condition, the current study also considered the PISA, similar to a previous study comparing patients with severe HF with an underlying ICM or DCM [31]. The PISA allows for the quantification of periodontal inflammation as a sum of inflamed periodontal pockets [32]. The periodontitis stage only reflects periodontal history (past destruction of periodontium). While PISA was presumed to be clinically more promising, no association was shown for PISA, either. Altogether, periodontitis appears not to be a risk indicator for (cultured) microbiological findings at the driveline of LVAD, but respective patients need increased attention in dental care. As proposed by Javed et al., sufficient protocols for dental treatment and prevention, including appropriate antimicrobial strategies, are needed [11]. Until now it has been unclear whether the presence of periodontitis has an influence on antibiotic treatment and respective outcomes in LVAD carriers. It is known that infections of the LVAD driveline are difficult to manage, often requiring a prolonged antibiotic treatment [3,4,5,6]. Therefore, potential risk predictors or parameters to adapt the antibiotic treatment could be relevant. While the current study did not confirm periodontal parameters to be associated with bacterial colonization, the potential relevance of oral inflammation remains unclear. Especially the high periodontitis prevalence in LVAD patients might obscure an effect. This might be an issue of potential relevance that should be addressed in future studies in the field.

This is the first study examining the associations between periodontitis severity as well as inflammation and microbiological colonization of the driveline of patients with an LVAD. The inclusion of 73 patients with severe heart diseases and LVAD treatment appears to be a strength of the study. However, the subgroups, which were compared in the current study, were quite small. Especially regarding specific (only cultured) bacterial species, no deeper analysis was possible due to the low case number. In this respect, the results of the current study must be seen as preliminary. Although all examinations were performed by calibrated dentists under standardized conditions, the retrospective character of the study is a methodological limitation. For a robust statement, a prospective design with a larger sample size would be needed. Similarly, the microbiological methodology is limited, as the applied cultivation method does not cover the common potential periodontal pathogens. In this context, microbiological samples from the oral cavity could be useful to add in further studies. Furthermore, disease-specific (driveline infection or not, localization of infection, severity of infection, laboratory values) as well as many patient-specific parameters, e.g., co-morbidities, smoking and cardiological data were not considered due to the limited sample size. In a future study with a larger sample size, a multivariate model would bring more strong conclusions. The absence of a healthy control group is not a limitation of the current study because the association between periodontitis and driveline colonization can only be examined in patients with an LVAD. However, in further studies, edentulous patients could be considered as a sort of “control group” (periodontitis vs. edentulous); in this study, edentulous patients were excluded because there were only a few respective edentulous participants available. For interpretation of the periodontal findings, the Fifth German Oral Health Study was used as a population-representative examination [26].

## 4. Conclusions

Within the limitations of the current study, periodontitis severity and inflammation are not associated with (cultured) microbiological findings at the driveline of patients with an LVAD. Although periodontitis appears not to be a risk indicator for driveline colonization, the high periodontal burden in this patient group underlines the need for their improved periodontal care.

## 5. Materials and Methods

This clinical retrospective cohort study was performed to examine potential associations between bacterial findings on LVAD systems and periodontal disease parameters. All examinations and analyses comply with the Declaration of Helsinki, and the ethics committee of the Medical Faculty of University of Leipzig has approved the study protocol (No: 414/16-ek). All participants provided their written informed consent for the current study.

### 5.1. Patients

Within this retrospective study, a total of 128 patients under treatment with an LVAD attending the Department for Cardiac Surgery at the Heart Center Leipzig between January 2016 and December 2018 were checked for their eligibility. The following inclusion criteria were mandatory for participation in the current analysis:

- Mean age of 18 years;
- Ability to provide informed consent at the time point of examination;
- Full oral examination within the 12 months prior to or after microbiology;
- Microbiological examination of the driveline in the study period.

The exclusion criteria were as follows:

- Clinical examination impossible due to worse general health status;
- Auto-immune diseases (e.g., rheumatoid arthritis);
- Infectious diseases (hepatitis A, B, C, tuberculosis, HIV);
- Pregnancy;
- Being edentulous.

Based on the patients’ records, all data from the oral examinations, which were performed within a previous cross-sectional study [9], were extracted. Furthermore, the findings from the microbiological analysis were recorded. As general health and cardiological data, smoking habits (smoker: currently smoking, former smoker: smoking within five years before examination, non-smoker: no smoking for at least five years), age and gender, body mass index (BMI), underlying heart diseases, co-morbidities and different disease/therapy-related parameters were collected from the medical records.

### 5.2. Microbiological Sample Collection and Analysis

The microbiology in the current study was based on the cultivation of respective bacteria. For microbiological analysis, patients were examined during their routine follow-up appointment. After removal of the closure cap of the driveline at the entry point on the skin surface, a swab was taken, placed in a sterile transportation tube, and sent to the laboratory for analysis. Thus, only one swab of the skin around the driveline was taken with a sterile stick. In the laboratory, the swabs were scratched out on agar plates and incubated at 37 °C for 72 h. Afterwards, a microscopic evaluation of the respective bacterial species was performed to identify the colonizing microbiota. Only bacteria that could be cultivated were considered for analysis.

### 5.3. Oral Examination

Three experienced and calibrated dentists performed the oral examinations under standardized conditions in the Department of Cardiac Surgery, as described previously [9]. One hour prior to oral examination, all patients received 2 g Amoxicillin as an antibiotic prophylaxis [33]. Based on the dental examination, the number of remaining teeth was recorded. All included patients underwent a comprehensive periodontal examination, including periodontal probing depth (PPD) and clinical attachment loss (CAL) at six measurement points per tooth with a periodontal probe (PCP 15, Hu-Friedy, Chicago, IL, USA). Moreover, the bleeding on probing (BOP) was recorded. Based on periodontal findings (PPD and CAL), the periodontitis severity (stage and grade) was determined according to the current classification within the available staging (stage I–IV) and grading (grade: A–C) matrix [34]: Stage I: interdental CAL max. 1–2 mm, Stage II: interdental CAL max. 3–4 mm, Stage III: interdental CAL max. ≥5 mm, periodontitis-related tooth loss ≤4 teeth, Stage IV: interdental CAL max. ≥5 mm, periodontitis-related tooth loss ≥5 teeth, and Grade A: bone loss/age <0.25, Grade B: bone loss/age 0.25–1.0 and/or smoking <10 cigarettes/day and/or diabetes mellitus with HbA1c <7.0%, Grade C: bone loss/age <1.0 and/or smoking ≥10 cigarettes/day and/or diabetes mellitus with HbA1c ≥7%.

Furthermore, the periodontal inflamed surface area (PISA) was determined based on the periodontal pockets with positive bleeding (BOP+) to quantify the surface of inflamed periodontal pockets [35].

### 5.4. Statistical Analysis

The statistical analysis was performed with SPSS for Windows, version 24.0 (SPSS Inc., Chicago, IL, USA). Normal distribution was tested with the Kolmogorov–Smirnov test, where mostly non-normal distribution was determined. The Mann–Whitney U test was used as a non-parametric test, while categorical data were analyzed with a chi-square or Fisher test, respectively. A two-sided significance testing was used for all tests. The significance level has been set at *p* < 0.05.

## Figures and Tables

**Table 1:** Patient characteristics.

Parameter		LVAD (*n* = 73)
Gender (female in % (*n*))		5.5% (4)
Age in years (mv ± sd)		60.92 ± 8.98
Smoking habits % (*n*)	smoker	12.3% (9)
	non-smoker	68.5% (50)
	former smoker	19.2% (14)
BMI (kg/m^2^)		28.37 ± 4.27
Underlying heart disease % (*n*)	dcm	47.9% (35)
	icm	50.7% (37)
	valvular insufficiency	56.2% (41)
	atrial fibrillation	47.9% (35)
	coronary heart disease	52.1% (38)
Co-morbidities % (*n*)	hypertension	75.3% (55)
	diabetes mellitus	50.7% (37)
	renal insufficiency	53.4% (39)
	adipositas	41.1% (30)
Driveline infection		30.1% (22)
Ejection fraction		24.33 ± 7.67
On waiting list for HTx		42.5% (31)
Time since LVAD implantation in months		32.00 ± 24.03
LVAD intension	BTT	41.1% (30)
	destination	34.2% (25)
	decision	23.3% (17)
	recovery	1.4% (1)
Urgency of LVAD implantation	emergency	17.1% (12)
	urgent	70.0% (49)
	elective	12.9% (9)

LVAD: left ventricular assist device, mv: mean value, sd: standard deviation, BTT: bridge to transplantation, BMI: body mass index, dcm: dilatative cardiomyopathy, icm: ischemic cardiomyopathy.

**Table 2:** Microbiological findings of LVAD patients.

Parameter		LVAD (*n* = 73)
Bacterial findings in general % (*n*)		80.8% (59)
Bacterial findings at day of oral examination % (*n*)		46.6% (34)
Bacterial 12 months prior/after oral examination % (*n*)		63.0% (46)
Bacterial species at day of oral examination % (*n*)	*Gram-negative bacilli*	6.8% (5)
	*Gram-positive cocci*	6.8% (5)
	*Staphylococcus aureus*	16.4% (12)
	*Staphylococcus epidermidis*	16.4% (12)
	*Staphylococcus lugdunensis*	1.4% (1)
	*Staphylococcus capitis*	4.1% (3)
	*Klebsiella pneumoniae*	2.7% (2)
	*Escherichia coli*	1.4% (1)
	*Enterococcus faecalis*	1.4% (1)
	*Corynebacterium amycolatum*	2.7% (2)
	*Proteus mirabilis*	2.7% (2)
	*Proteus volgaris*	1.4% (1)
	*Seratina marcescens*	1.4% (1)
	*Enterobacter cloace*	2.7% (2)
	*Achromabacter xylosoxidans*	1.4% (1)
	*Stenotrophomonas maltophilia*	2.7% (2)
	*Pseudomonas aeroginosa*	1.4% (1)
	*Dermabacter hominis*	1.4% (1)

LVAD: left ventricular assist device.

**Table 3:** Oral health conditions of included LVAD patients.

Parameter		LVAD (*n* = 73)
Number of remaining teeth (mv ± sd)		18.99 ± 7.58
Number of teeth CAL ≥ 5 mm (mv ± sd)		5.37 ± 5.13
Number of teeth PPD ≥ 5 mm (mv ± sd)		6.37 ± 6.80
CAL mean (mv ± sd)		3.08 ± 1.22
PPD mean (mv ± sd)		3.03 ± 0.76
BOP (%) mean (mv ± sd)		21.62 ± 20.02
Periodontitis stage % (*n*)	I	2.7% (2)
	II	16.4% (12)
	III	23.3% (17)
	IV	57.5% (42)
Grade % (*n*)	A	0
	B	45.2% (33)
	C	54.8% (40)
PISA (mv ± sd)		284.78 ± 352.29

mv: mean value, sd: standard deviation, PPD: periodontal probing depth, CAL: clinical attachment loss, BOP: bleeding on probing, PISA: periodontal inflamed surface area.

**Table 4:** Association between bacterial findings in general and periodontal health parameters.

Periodontal Parameter		Bacterial Findings (*n* = 59)	No Bacterial Findings (*n* = 14)	*p*-Value
PISA (mv ± sd)		286.10 ± 372.56	279.24 ± 260.88	0.75 *
PPD mean (mv ± sd)		3.04 ± 0.76	3.00 ± 0.82	0.88 *
CAL mean (mv ± sd)		3.12 ± 1.18	2.93 ± 1.40	0.50 *
BOP mean (mv ± sd)		21.32 ± 20.02	22.86 ± 20.74	0.92 *
Periodontitis stage % (*n*)	I	3.4% (2)	0	0.86 **
	II	15.3% (9)	21.4% (3)	
	III	23.7% (14)	21.4% (3)	
	IV	57.6% (34)	57.1% (8)	
Periodontitis grade % (*n*)	B	47.5% (28)	35.7% (5)	0.55 ***
	C	52.5% (31)	64.3% (9)	

mv: mean value, sd: standard deviation, PPD: periodontal probing depth, CAL: clinical attachment loss, BOP: bleeding on probing, PISA: periodontal inflamed surface area, * Mann–Whitney U test, ** Chi-square test, *** Fisher test.

**Table 5:** Association between bacterial findings on the day of oral examination and periodontal health parameters.

Periodontal Parameter		Bacterial Findings (*n* = 34)	No Bacterial Findings (*n* = 39)	*p*-Value
PISA (mv ± sd)		273.53 ± 232.29	294.59 ± 433.68	0.56 *
PPD mean (mv ± sd)		3.02 ± 0.72	3.04 ± 0.81	0.97 *
CAL mean (mv ± sd)		3.00 ± 1.15	3.15 ± 1.29	0.66 *
BOP mean (mv ± sd)		19.15 ± 14.45	23.77 ± 23.85	0.83 *
Periodontitis stage % (*n*)	I	5.0% (2)	0	0.33 **
	II	11.8% (4)	20.5% (8)	
	III	26.5% (9)	20.5% (8)	
	IV	55.9% (19)	59.0% (23)	
Periodontitis grade % (*n*)	B	47.1% (16)	43.6% (17)	0.82 ***
	C	52.9% (18)	56.4% (22)	

mv: mean value, sd: standard deviation, PPD: periodontal probing depth, CAL: clinical attachment loss, BOP: bleeding on probing, PISA: periodontal inflamed surface area, * Mann–Whitney U test, ** Chi-square test, *** Fisher test.

**Table 6:** Association between bacterial findings 12 months prior to/after oral examination and periodontal health parameters.

Periodontal Parameter		Bacterial Findings (*n* = 46)	No Bacterial Findings (*n* = 27)	*p*-Value
PISA (mv ± sd)		292.66 ± 400.11	271.36 ± 257.50	0.88 *
PPD mean (mv ± sd)		3.03 ± 0.76	3.03 ± 0.78	0.95 *
CAL mean (mv ± sd)		3.09 ± 1.17	3.07 ± 1.33	0.78 *
BOP mean (mv ± sd)		21.52 ± 20.52	21.78 ± 19.51	0.92 *
Periodontitis stage % (*n*)	I	4.3% (2)	0	0.71 **
	II	17.4% (8)	14.8% (4)	
	III	21.7% (10)	25.9% (7)	
	IV	56.5% (26)	59.3% (16)	
Periodontitis grade % (*n*)	B	47.8% (22)	40.7% (11)	0.63 ***
	C	52.2% (24)	59.3% (16)	

mv: mean value, sd: standard deviation, PPD: periodontal probing depth, CAL: clinical attachment loss, BOP: bleeding on probing, PISA: periodontal inflamed surface area, * Mann–Whitney U test, ** Chi-square test, *** Fisher test.

## Data Availability

Data are available from the corresponding author upon reasonable request.
